# Wernicke Encephalopathy and Possible Myelopathy Following Removal of an Intragastric Balloon: A Rare Complication in a Bariatric Patient

**DOI:** 10.7759/cureus.93156

**Published:** 2025-09-24

**Authors:** Ibrahim A Mohamed, Nafisa E Mohammed, Mathab Adam, Sama Rahma, Iman Jamal, Jamal Sajid

**Affiliations:** 1 Internal Medicine, Hamad Medical Corporation, Doha, QAT; 2 Medicine, Tbilisi State Medical University, Tbilisi, GEO; 3 Internal Medicine, Hamad General Hospital, Doha, QAT

**Keywords:** bariatric procedure, igb, intragastric balloon, thiamine deficiency, vitamin b1 deficiency, wernicke encephalopathy

## Abstract

Wernicke encephalopathy (WE) is a neurological emergency caused by thiamine (vitamin B1) deficiency. WE is typically diagnosed clinically in high-risk patients presenting with ophthalmoplegia, ataxia, and confusion. Thiamine deficiency is most often seen in people who consume excessive alcohol, but it can also occur in other conditions, such as chronic malnutrition, prolonged vomiting, or after bariatric procedures. Inadequate or delayed treatment can result in irreversible Korsakoff psychosis or, in severe cases, death. We report the case of a 25-year-old male patient who never consumed alcohol and underwent intragastric balloon (IGB) insertion for obesity management. Several months post-procedure, he experienced severe nausea and vomiting, necessitating the removal of the IGB. Within a couple of days of IGB removal, the patient developed dizziness and ophthalmoplegia. A clinical diagnosis of WE was established, and prompt administration of high-dose intravenous thiamine led to notable clinical improvement. The diagnosis of WE was subsequently confirmed upon MRI of the head. In addition to the manifestations of WE, the patient exhibited signs indicative of multilevel myelopathy. This report highlights a rare case of WE with possible myelopathy following IGB removal, a non-malabsorptive procedure, in a young male patient who never consumed alcohol.

## Introduction

Obesity, as a general term, is defined as an excess of body fat stores that have potentially negative impacts on one's health. Endoscopic intragastric balloon (IGB) therapy has emerged as an effective temporary alternative to pharmacological treatment for obesity. It is recommended by major gastroenterology associations as it provides greater efficacy with lower risks than conventional surgical procedures. This effectiveness is particularly evident when used in conjunction with lifestyle modifications [1].

Numerous studies highlight nutritional deficiencies in vitamins, minerals, and trace elements as a major drawback of bariatric surgical techniques [2]. Wernicke encephalopathy (WE) is an acute neurological disorder that results from vitamin B1 (thiamine) deficiency and consequent failure of cellular energy production. This can cause cytotoxic edema, oxidative stress, and selective neuronal loss in the mammillary bodies, dorsomedial thalami, periaqueductal grey matter, and cerebellar vermis [3]. Although WE is known to occur following malabsorptive operations, few isolated cases have been reported in association with IGB insertion or removal, demonstrating that follow-up is warranted in apparently non-malabsorptive operations [4].

Despite the integrity of the absorptive jejunal surface post balloon insertion, the supply-demand mismatch created by vomiting, reduced intake, rapid weight loss, and dextrose infusions can exhaust thiamine stores within days to weeks. This leads to deficiency and, in severe cases, WE [5].

Like the concern over diminished micronutrients, biochemical liver injury with balloon therapy is also common but usually a transient phenomenon; pooled values show median alanine-aminotransferase elevations of around two times the upper limit in the first month. Nevertheless, no evidence that acute WE may be associated with marked aminotransferase elevation in the same patient has ever been recorded [6].

In this study, we report the case of an obese 25-year-old male patient with an initial BMI of 36 kg/m^2^ who developed elevated transaminase levels and was diagnosed with WE following the removal of an endoscopic IGB, emphasizing a preventable yet life-threatening complication of endoscopic bariatric therapy.

## Case presentation

A 25-year-old male patient with class II obesity (BMI of 36 kg/m^2^; weight: 111 kg, height: 175 cm) and a history of controlled hypothyroidism was electively admitted for the removal of an IGB. His medical and surgical histories were otherwise unremarkable.

Twenty months prior to admission, he had his first IGB procedure, during which a Heliosphere balloon (Helioscopie, Vienne, France) was placed for seven months. A minimal weight loss of 8 kg was achieved, reducing his weight from 108 kg to 100 kg, and the perioperative course was uneventful. Six months later, he underwent a second IGB placement using an Endalis balloon (Endalis, Brignais, France). This procedure was complicated by severe epigastric pain and vomiting, requiring the balloon to be removed after seven months. Two days after the second IGB removal, the patient developed dizziness and unsteadiness while continuing to experience gastrointestinal symptoms.

**Figure 1:**
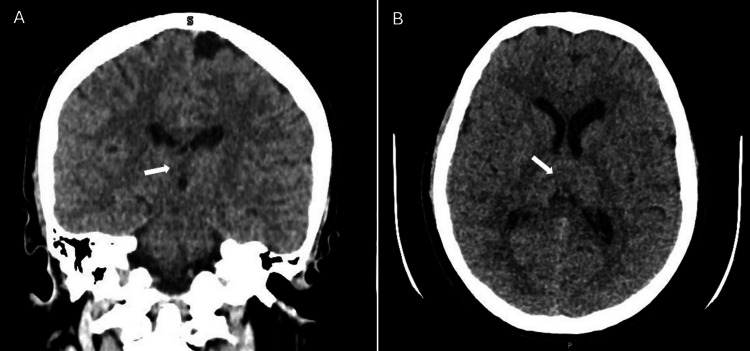
CT images of the patient’s brain. Coronal (A) and transverse (B) views of brain CT showing a hypodense area in the floor of the third ventricle (white arrows).

**Figure 2:**
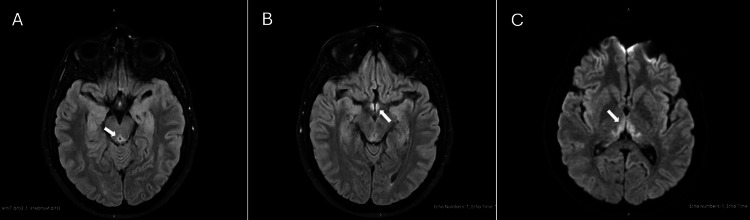
Magnetic resonance images of the patient’s brain. MRI brain showing high-signal intensity alterations in the periaqueductal gray (A, white arrow), the mamillary body (B, white arrow), and the bilateral thalami (C, white arrow).

**Figure 3:**
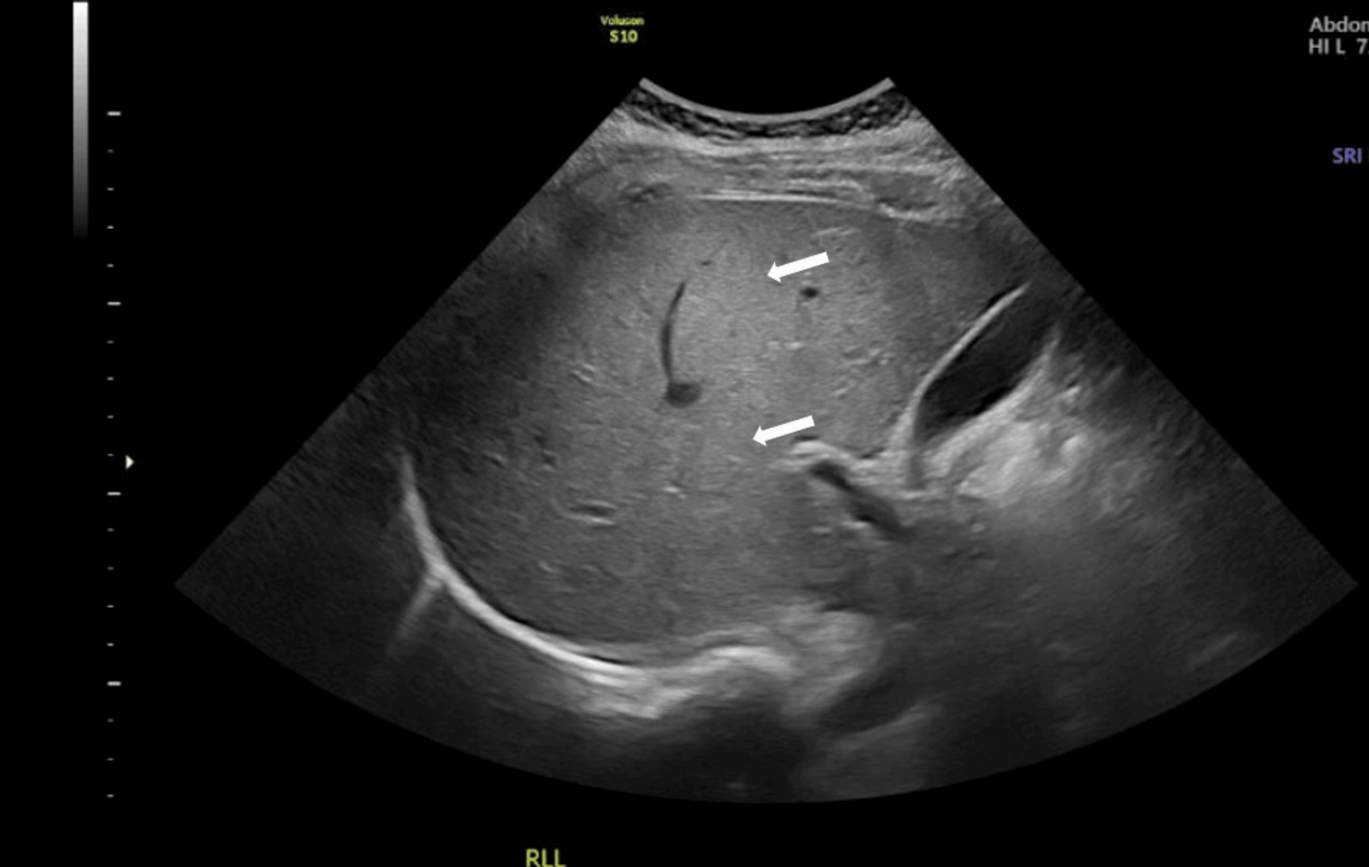
Ultrasound image of the patient’s liver. Ultrasound of the liver showing diffuse hyperechogenicity (white arrows) consistent with moderate hepatic steatosis.

Examination revealed bilateral vertical and horizontal nystagmus on lateral gaze, dysmetria during the finger-to-nose test, a wide-based ataxic gait, and a positive Hoffman sign on the left. Bilateral lower limb osteotendinous reflexes were diminished. In view of these neurological changes, a clinical diagnosis of WE was made. High-dose IV thiamine was immediately started to prevent further neurological sequelae. Laboratory tests (Table 1) showed abnormal liver function tests (aspartate transaminase (AST): 207 U/L, alanine transaminase (ALT): 630 U/L, alkaline phosphatase (ALP): 146 U/L, total bilirubin: 40 μmol/L, and direct bilirubin: 33 μmol/L). The remaining blood work, including serum vitamin levels, was normal. Upon further investigations, a CT scan of the brain demonstrated a hypodense area in the floor of the third ventricle (Figure 1). An MRI of the brain revealed high T2/fluid attenuated inversion recovery (FLAIR) hyperintensities in the periaqueductal gray, mammillary bodies, tectal plate, and bilateral thalami, confirming our initial diagnosis of WE (Figure 2). Abdominal ultrasound showed moderate hepatic steatosis (Figure 3).

**Table 1:** Laboratory testing results. Values marked with (*) were taken after starting multivitamins and thiamine infusions. ↑: higher than normal range; ↓: lower than normal range; WBC: white blood cells; AST: aspartate transaminase; ALT: alanine transaminase

Investigation	Results with units	Normal range
WBC	7.8 x10³/μL	4.0-10.0
Neutrophils	4.3 x10³/μL	2.0-7.0
Lymphocytes	2.3 x10³/μL	1.0-3.0
Hemoglobin	13.9 g/dL	13.0-17.0
Platelets	312 x10³/μL	150-410
Urea	2.5 mmol/L	2.5-7.8
Creatinine	70 μmol/L	62-106
Sodium	145 mmol/L	133-146
Potassium	3.8 mmol/L	3.5-5.3
Bilirubin (Total)	40 μmol/L ↑	0-21
Bilirubin (Direct)	33 μmol/L ↑	0-5
Albumin	32 g/L ↓	35-50
Alkaline phosphatase	146 U/L ↑	40-129
ALT	630 U/L ↑	0-50
AST	207 U/L ↑	0-50
Vitamin A	3.3 μmol/L * ↑	1.0-2.1
Vitamin B1	459 nmol/L * ↑	66-201
Vitamin B6	55 nmol/L *	22-121
Vitamin B12	379 pmol/L	145-569
Folate	7 nmol/L ↓	10-70
Copper	15.2 μmol/L	11.8-22.8
Zinc	12.3 μmol/L	10.1-16.8

Patient received high-dose IV thiamine 500 mg three times daily for three days, then 500 mg twice daily for another three days, and finally 250 mg once daily for a week. Clinically, his symptoms improved over the first few days, and he became ambulatory with support, although external ophthalmoplegia and dizziness persisted.

Following this limited improvement, the patient began experiencing paraparesis and sensory deficits. Further examination revealed variable sensory levels at D9-D10, then at D12-L1, and subsequently in the low dorsal region over the following days. Although an MRI of the spine was arranged, the patient refused it. These clinical findings raised concerns about coexisting nutritional myelopathy, such as subacute combined degeneration, despite normal vitamin B12 levels.

The clinical presentation suggested WE with possible spinal cord involvement, likely caused by nutritional deficiencies. This is a rare but severe complication of bariatric procedures, highlighting the importance of early evaluation and treatment of related vitamin deficiencies.

## Discussion

Thiamine is a water-soluble vitamin stored in small amounts in the liver. Its deficiency can cause various heart and brain-related complications. Thiamine deficiency results from several factors, including reduced dietary intake, malabsorptive conditions, and increased urine losses due to alcohol use or medication side effects. Severe deficiency can result in beriberi and Wernicke-Korsakoff syndrome, both of which are seen in conditions like excessive alcohol consumption, celiac disease, HIV/AIDS, and post-bariatric surgeries [7]. Mortality rates in untreated cases can reach nearly 20%, and among survivors, almost 85% may develop Korsakoff psychosis, a chronic condition characterized by profound memory impairment, disorientation, and confabulation [8].

WE is an acute neurological condition resulting from thiamine deficiency. Although chronic alcohol use is the most common cause, other factors such as bowel surgeries, chemotherapy use, and hyperemesis gravidarum have also been reported [9-11].

Bariatric surgeries, such as Roux-en-Y and sleeve gastrectomy, are often associated with nutritional deficiencies because they can cause early satiety and malabsorption of nutrients. In contrast, restrictive techniques like IGB placement are less frequently associated with these complications [12,13]. Our case describes a rare instance of WE following IGB removal. IGB is a restrictive, non-anatomy-altering weight-loss procedure.

Typically, WE presents with the classic triad of ophthalmoplegia, ataxia, and mental status changes, although this triad appears in only about 30% of patients [14]. Early symptoms such as dizziness, fatigue, or gastrointestinal discomfort are often vague and non-specific [15]. In our patient, neurological examination revealed bilateral nystagmus, dysmetria, ataxia, and hypoactive reflexes, findings that align with the clinical spectrum of WE. MRI findings of T2/FLAIR hyperintensities in the periaqueductal gray, mammillary bodies, tectal plate, and thalami further supported the diagnosis.

Unlike typical patients with a history of alcohol use or gastric bypass surgeries, our patient developed WE following severe and persistent vomiting after IGB removal. Vomiting is a well-known cause of thiamine depletion, with body stores potentially exhausted within three to six weeks. A cohort study involving 957 patients who underwent post-bariatric procedures found that 90% of those experiencing vomiting had a vitamin B1 deficiency [14]. This study highlights that even restrictive procedures like IGB, which are often considered safer, can result in significant nutritional deficiencies.

A systematic review from 2007 analyzed 32 case reports involving WE patients aged 23 to 55 years. The onset of WE occurred anywhere from two weeks to 78 weeks after bariatric surgery, with most cases occurring between four and 12 weeks [16]. Only a few case reports in the literature have directly linked WE to IGB. In one report, the patient developed WE just nine days after balloon insertion due to vomiting, while in another case, symptoms appeared three months after the procedure [13,17]. In both instances, the balloon was removed following the appearance of neurological symptoms. Our patient, however, began experiencing neurological symptoms two days after gastric balloon removal. In most cases in the literature, the patient developed symptoms of WE while the IGB was in place, whereas our patient experienced symptoms after IGB removal.

In addition to the classic features of WE, our patient experienced worsening weakness in the lower limbs and sensory deficits with shifting thoracic sensory levels, raising suspicion of a concurrent nutritional myelopathy. Although vitamin B12 levels were normal, a functional B12 deficiency or other micronutrient deficiencies (e.g., copper, folate) may have played a role. Spinal cord involvement is uncommon in typical WE, indicating a broader neurological injury related to malnutrition.

Furthermore, the patient showed elevated transaminase and bilirubin levels. Although these findings are not typical for isolated WE, they may suggest hepatic stress caused by prolonged vomiting, malnutrition, or possible refeeding syndrome.

In summary, this case illustrates several important deviations from the typical presentation of WE. First, it occurred in a young, non-alcoholic male patient following a restrictive, non-surgical procedure. Second, it involved spinal cord findings suggestive of subacute combined degeneration despite normal B12 levels. Third, it was associated with substantial hepatic dysfunction, likely due to prolonged vomiting and nutritional deficiency. Lastly, it contributes to the literature on WE in male patients, as most bariatric interventions and their complications are reported in female patients.

These findings emphasize the importance of maintaining a high index of suspicion for WE in any patient presenting with gastrointestinal symptoms and neurological changes, regardless of the type of bariatric intervention. Early recognition, prompt neuroimaging, and immediate thiamine replacement are essential to prevent irreversible complications.

## Conclusions

Most of the literature highlights the strong association between bariatric surgical procedures and the development of WE as a potential complication. However, the case presented here demonstrates that WE can also happen without surgery, following non-invasive weight-loss methods like IGB therapy. In light of this, we believe that patients undergoing any bariatric procedure, especially those with ongoing vomiting, should be carefully monitored and timely assessed for neurological symptoms and signs, as well as vitamin deficiencies. We recommend routine thiamine level screening for all patients who underwent a bariatric IGB procedure. Early treatment is crucial for improving outcomes and preventing the progression to Korsakoff syndrome.
